# Barriers to adequate pain control among women with cervical cancer: exploring unmet pain control needs in Ghana

**DOI:** 10.1016/j.xagr.2022.100065

**Published:** 2022-06-18

**Authors:** Sarah G. Bell, Adu Appiah-Kubi, Thomas O. Konney, Augustine Tawiah, Samuel Yost, Emily K. Kobernik, Emma R. Lawrence

**Affiliations:** a Department of Obstetrics and Gynecology, University of Vermont, Burlington, VT; b Department of Obstetrics and Gynaecology, School of Medicine, University of Health and Allied Sciences, Ho, Ghana; c Department of Obstetrics and Gynecology, Komfo Anokye Teaching Hospital, Kumasi, Ghana; d Department of Obstetrics and Gynecology, University of Michigan, Ann Arbor, MI

**Keywords:** cancer pain, cancer pain severity, cervical cancer, gynecologic cancer, low- and middle-income country, pain attitudes, pain management, pain medications, sub-Saharan Africa

## Abstract

**BACKGROUND:**

Pain because of cervical cancer is a significant health issue globally, especially in women with advanced disease. However, little is known about unmet needs for pain control in low-resource settings where the burden of cervical cancer is the greatest.

**OBJECTIVE:**

This study aimed to quantify the level of pain that women with cervical cancer in Ghana experience, explore attitudes toward pain and pain medications, and determine the barriers to adequate pain control.

**STUDY DESIGN:**

A cross-sectional survey was conducted on 100 adult women with a histopathologic diagnosis of cervical cancer presenting for care at Komfo Anokye Teaching Hospital in Ghana. In addition, a descriptive analysis was conducted among all participants and the subgroup of women who reported pain but did not report pain medication use.

**RESULTS:**

Among 100 participants with cervical cancer, the mean age was 59.5 years, and the median parity was 6.0 (interquartile range, 5.0–6.0); moreover, most participants presented with inoperable stage II or greater cervical cancer (99 of 100 [99%]). Of 100 participants, 80 (80%) had pain caused by their cervical cancer, with more than half (51 of 100 [51%]) rating their pain as a 3, 4, or 5 on a 5-point scale. Most participants reported pain significant enough to impact their sleep (58 of 99 [58.6%]) and their ability to carry out daily activities (54 of 100 [54%]). Furthermore, 55 of 100 participants (55%) took pain medications in the last week; however, only 5 of 54 participants (9.3%) reported complete improvement in their pain, and most participants (30 of 54 [55.6%]) felt they needed a stronger pain medication. Barriers to adequate pain control included the healthcare provider's focus on pain, with 14.1% of women reporting that their healthcare providers never asked about their pain (14 of 99 [14.1%]). In addition, participants’ attitudes toward pain control demonstrated that 34 of 95 participants (35.8%) believed that they should be able to tolerate their cervical cancer pain without medication. Among participants who ever took pain medication, 16 of 58 (27.6%) were bothered that they took pain medication, and 19 of 58 (32.7%) were concerned that they used too much medication. Most participants were able to afford (51 of 58 [88%]) and access (56 of 58 [96.6%]) pain medications and did not worry their supply would run out (56 of 58 [96.6%]).

**CONCLUSION:**

Most patients had significant pain because of cervical cancer, and many of them endorsed needing more pain medications than what they were prescribed. The etiologies of the unmet need for pain control included missed opportunities to discuss pain control at clinic visits and patients’ attitudes toward pain management. Financial and access barriers to obtaining pain medications were minimal.

**AJOG Global Reports at a Glance**

**Why was this study conducted?**

This study aimed to describe unmet needs for pain control among women with cervical cancer in Ghana by quantifying the level of pain experienced, exploring attitudes toward pain and pain medications, and determining barriers to adequate pain control.

**Key findings**

Here, 80% of women had pain from their cervical cancer, and in most cases, the pain was significant enough to impact their sleep and daily activities; moreover, 14.1% of women reported that their healthcare providers “never” asked about their pain, and 35.8% of women believed they should be able to tolerate their pain without medication.

**What does this add to what is known?**

Our findings have challenged the assumption that financial and geographic barriers are the primary drivers of the unmet need for pain control in sub-Saharan Africa and highlighted the importance of women's attitudes toward pain control and the role of healthcare providers in prioritizing pain management.

## Introduction

Cervical cancer primarily affects women in low- and middle-income countries (LMICs) where routine screening programs for premalignant cervical lesions are not available or ineffective. Approximately 100,000 new cases of cervical cancer are diagnosed in sub-Saharan Africa each year, with more than 3000 of those occurring in Ghana.^1^ The rate of cervical cancer screening with the Papanicolaou test and/or human papillomavirus testing is only 2.8% in Ghana,^1^ compared with 93% of women in the United States having at least 1 lifetime Papanicolaou test.^2^ As screening rates in Ghana are low, most new diagnoses are in patients presenting at advanced stages. A study from the 2 largest metropolitan regions in Ghana found that only 5.2% of cases of cervical cancer were operable stage I disease, with the remaining 94.8% of patients presenting with advanced inoperable disease.^3^ Therefore, more than 2000 women die in Ghana each year because of cervical cancer.^1^

Globally, many women with cervical cancer, especially those with locally advanced and metastatic disease, require significant prescription pain medications.^4^ The degree of pain varies by timing of diagnosis and treatment course.^1^ Even after treatment is completed, a substantial proportion of survivors of cervical cancer will continue to experience pain and reduced quality of life.^5^^,^^6^ Studies on cervical cancer pain in LMICs are limited, with existing studies demonstrating that patients in low-resource settings face challenges obtaining and affording pain medications^7^ and often rely on traditional and complementary medicine.^8^ The roles of patients’ attitudes toward pain and healthcare providers’ focus on pain control are largely unknown.

In LMICs, the availability of specialized facilities and healthcare providers to care for patients with cervical cancer is limited.^9^ The Komfo Anokye Teaching Hospital (KATH) in Kumasi, Ghana, offers a unique setting to study cervical cancer pain. At KATH, hundreds of women with new diagnoses of cervical cancer are seen each year, with most women presenting with locally advanced or metastatic cervical cancer.^3^ Thus, pain may be a significant burden for these patients. However, there is little information known about pain and pain treatment among women with cervical cancer in Ghana. Our study aimed to close this gap by surveying women with cervical cancer at KATH to quantify the level of pain experienced, identify the type and frequency of pain medications used, explore attitudes toward pain and pain medications, and determine the barriers to achieving pain control.

## Materials and Methods

We conducted a cross-sectional survey to explore the levels of pain among women with cervical cancer, attitudes toward pain medications and pain control, barriers to adequate pain control, and the role of healthcare providers in managing pain control. We collected data from October 2020 to July 2021. Ethical approval was granted by institutional review boards at the KATH (IRB/AP/112/20) and the University of Michigan (HUM00174977).

The study site was KATH, Ghana's second-largest teaching hospital, which serves as a tertiary referral hospital for patients throughout central Ghana. Unique in LMICs, KATH has a dedicated gynecologic oncology unit and is 1 of only 2 gynecologic oncology fellowship training centers in Ghana. The division manages approximately 250 women with a new cervical cancer diagnosis each year, with most cases being referrals from surrounding hospitals. The inclusion criteria were women with a histopathologic diagnosis of cervical cancer, receiving care at the gynecologic oncology clinic at KATH, being ≥18 years old, and fluency in English or Twi. At KATH, patients are seen for an initial gynecologic oncology clinic visit for a new patient history, examination, and biopsy. Once biopsy results have been finalized by pathology, patients are seen for a second clinic visit to discuss the diagnosis and stage of cervical cancer and to plan treatment. The participants were recruited at their second clinic visit; at that time, patients had a confirmed histopathologic diagnosis of cervical cancer and a clinical stage, but most had not yet initiated treatment.

Recruitment was conducted at the gynecologic oncology clinic. A list of eligible participants was determined before the start of each clinic through a review of the anticipated clinic schedule with the gynecologic oncology healthcare provider. A trained Ghanaian health research assistant carried out the recruitment, written informed consent, and data collection in either English or Twi, per participant preference. An incentive of cellphone credit valued at 3 US dollars was provided for participation. The survey was verbally administered to participants, and their responses were recorded by the research assistant.

The survey consisted of 6 sections. Section 1 consisted of demographic information, including age, parity, education, and income. Section 2 focused on quantitative assessment of daily pain and the impact of pain on daily activities. Pain scale questions were a combination of the validated Brief Pain Inventory (BPI) and the African Palliative Care Association African Palliative Outcome Scale.^10^, ^11^, ^12^ The BPI has been validated in patients with cancer and is used widely in both research and clinical settings. The African Palliative Outcome Scale has been validated in the African clinical setting and was used to guide question development. The final questionnaire used in our study was developed as a combination of these 2 scales with local expert opinion via the gynecologic oncologists at KATH. Section 3 focused on the use of pain medications, including type and frequency. In Kumasi, Ghana, the only readily available pain medications are diclofenac (rectal suppository nonsteroidal anti-inflammatory drugs), liquid morphine, tramadol, acetaminophen, and meperidine. Section 4 focused on attitudes surrounding pain and the use of pain medications. Section 5 focused on barriers to pain control, including questions about the cost and accessibility of pain medications. Section 6 focused on the perceived provider counseling and its role in pain control. Questions were a combination of multiple choice and Likert scales. In addition, relevant clinical information was collected from participants’ medical records, including cervical cancer stage and histopathologic diagnosis.

Survey data were entered in Research Electronic Data Capture and downloaded into SAS (version 9.4; SAS Institute Inc, Cary, NC) for analysis. Frequencies and proportions were calculated for categorical data. The normality of continuous variables was determined by assessing skewness and kurtosis and the Shapiro-Wilk test. Means and standard deviations were used for normally distributed continuous variables, and medians and interquartile ranges (IQRs) were used for nonnormally distributed continuous variables. A descriptive subgroup analysis was performed among women who reported pain in the last week but did not report pain medication use.

## Results

Among 102 sequential patients eligible for inclusion, 2 left the clinic before completing their clinical care and thus were not recruited to participate. All 100 remaining patients agreed to participate and completed the survey. The mean age of participants was 59.5 years, the median parity was 6.0 (IQR, 5.0–6.0), and most were either married (33 of 100 [33.0%]) or widowed (47 of 100 [47.0%]) (Table 1). Most participants (89 [89.0%]) earned <500 cedis per month (equivalent to approximately USD $80). All but 1 participant presented with inoperable stage II or greater cervical cancer (99 of 100 [99.0%]). Most patients had histopathology consistent with squamous cell carcinoma (87 of 98 [88.8%]) and had not yet initiated a treatment regimen (95 of 97 [97.9%]).

**Table 1:** Participant demographics

Characteristic	n (%), mean±SD, or median (IQR)
Age (y)	59.5±14.3
Parity	6.0 (5.0–6.0)
Marital status (n=100)	
Single	4 (4.0)
Married	33 (33.0)
Widowed	47 (47.0)
Divorced	16 (16.0)
Occupation (n=100)	
No work outside the home	32 (32.0)
Retired	22 (22.0)
Farmer	18 (18.0)
Trader	24 (24.0)
Professional work	1 (1.0)
Other	3 (3.0)
Highest education completed (n=96)	
None	48 (50.0)
Primary	32 (33.3)
Secondary	16 (16.7)
Monthly income (n=100)	
<500 cedis^a^	89 (89.0)
≥500 cedis^a^	11 (11.0)
Cervical cancer stage (n=100)	
I	1 (1.0)
II	20 (20.0)
III	71 (71.0)
IV	7 (7.0)
Unknown	1 (1.0)
Cervical cancer histopathology (n=98)	
Squamous cell carcinoma	87 (88.8)
Adenocarcinoma	7 (7.1)
Unknown or other	4 (4.1)
Current status of cervical cancer (n=97)	
New diagnosis, no treatment yet	95 (97.9)
Completed radiation therapy, now with recurrence	2 (2.1)

*IQR*, interquartile range; *SD*, standard deviation.

a Conversion rate: 62 US dollars.

**Figure 1:**
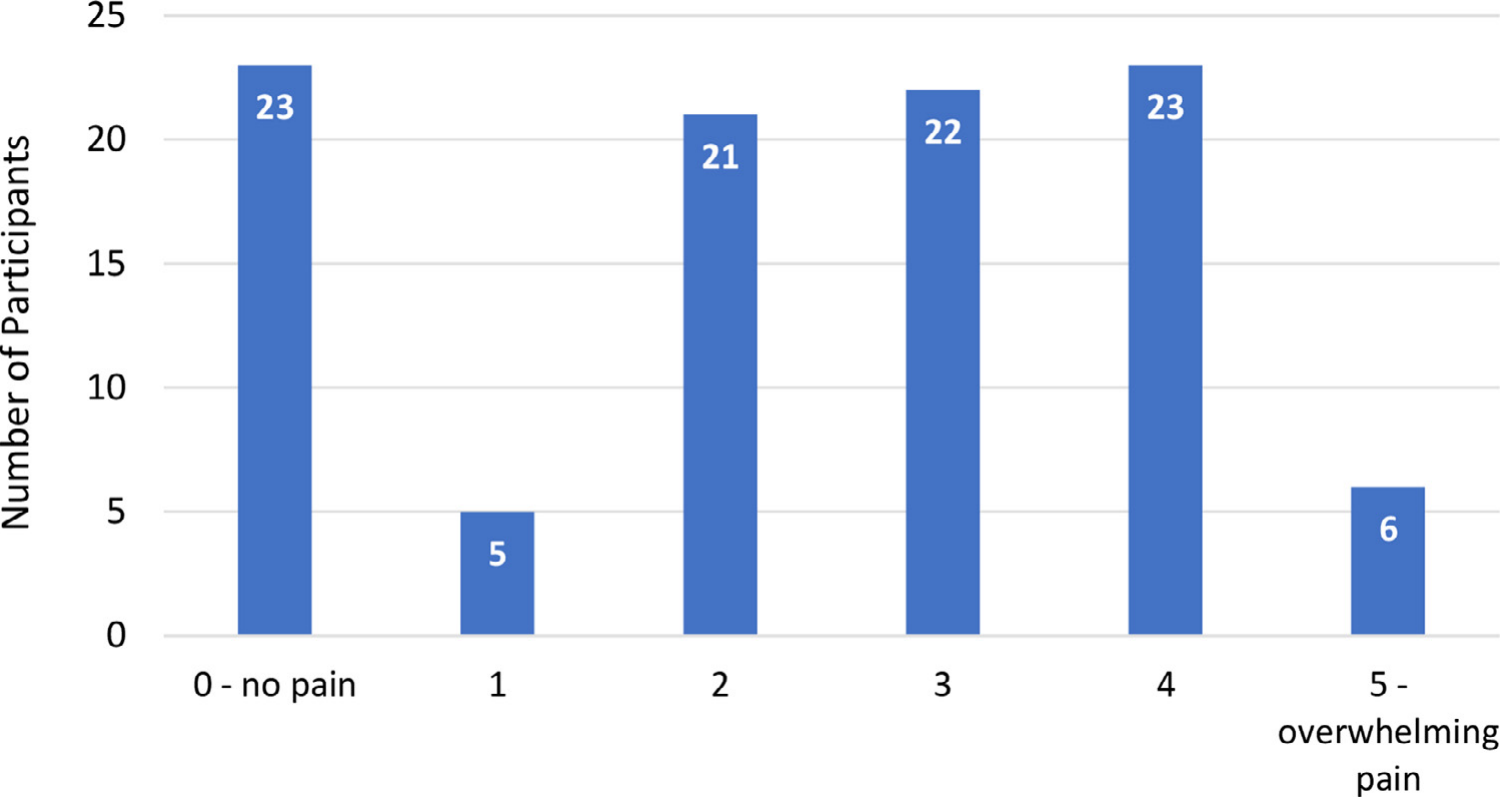
Level of cervical cancer pain experienced in the past week

Of 100 participants, 80 (80%) endorsed pain because of their cervical cancer (Table 2). On a 5-point scale from 0 (no pain) to 5 (overwhelming pain), more than half of the participants (51 of 100 [51.0%]) rated their pain as 3, 4, or 5 (Figure 1). Most participants felt their cervical cancer pain was more severe than the pain they experienced with childbirth (50 of 80 [62.5%]). Most reported that their pain negatively influenced their sleep (58 of 99 [58.6%]) and their ability to carry out daily activities (54 of 100 [54.0%]). Among those who were limited in their daily activities because of pain, most participants (40 of 54 [74%]) received help from their children.

**Table 2:** Experience with cervical cancer pain

Pain variable	n (%)
Presence of pain caused by cervical cancer (n=100)	
No	20 (20.0)
Yes	80 (80.0)
Intensity of cervical cancer pain compared with intensity of childbirth pain	
Cancer pain is less than childbirth pain	30 (37.5)
Cancer pain is more than childbirth pain	50 (62.5)
Presence of cervical cancer pain that affects daily activities (n=100)	
No	46 (46.0)
Yes	54 (54.0)
Source of help for daily activities affected by pain	
Husband	3 (5.6)
Children	40 (74.1)
Other	11 (20.4)
Presence of cervical cancer pain that affects sleep (n=99)	
No	58 (58.6)
Yes	41 (41.4)

Bell. Barriers to cervical cancer pain control in urban Ghana. Am J Obstet Gynecol Glob Rep 2022.

**Figure 2:**
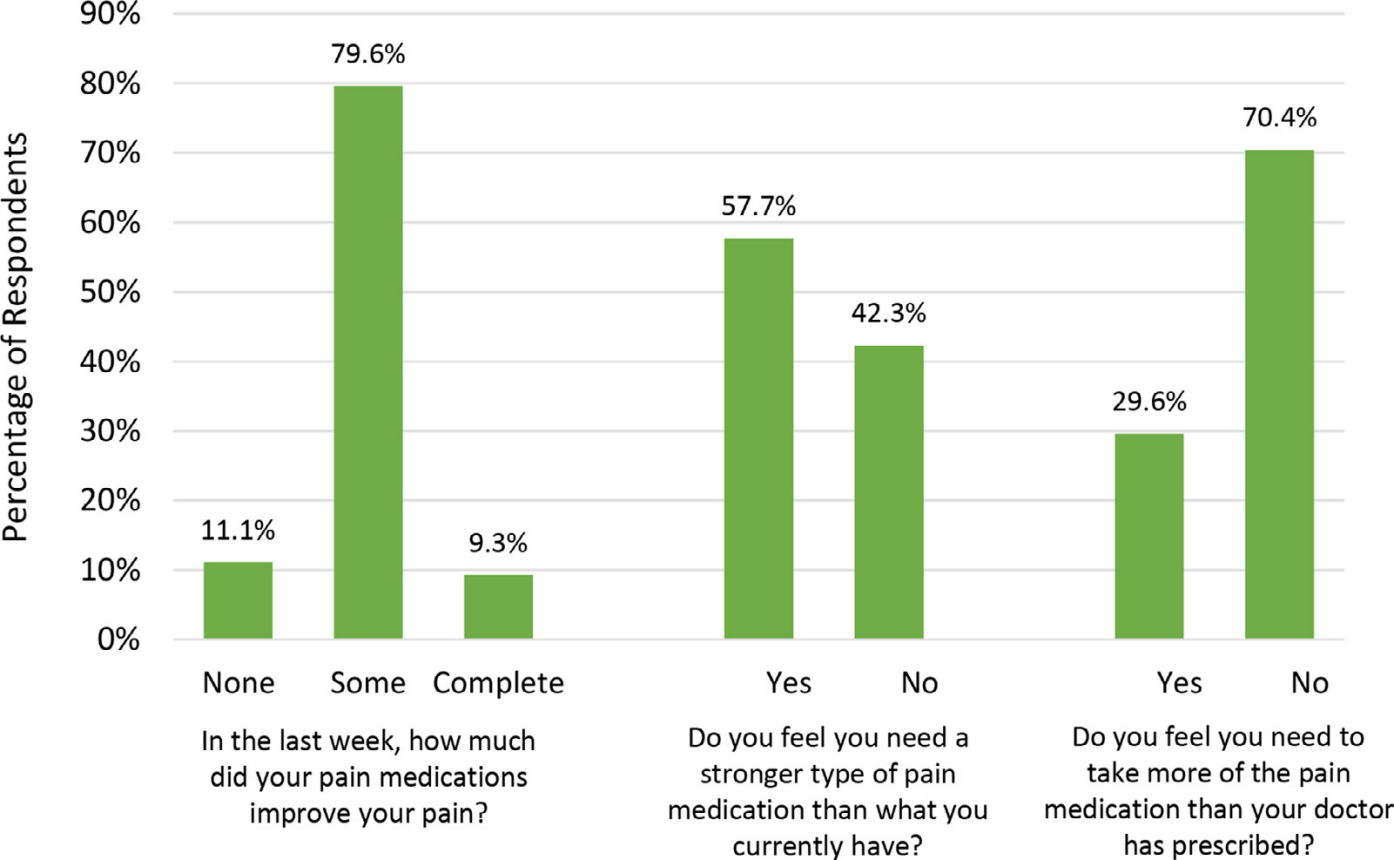
Improvement in pain among participants taking pain medications

Regarding the use of pain medications, most participants (55 of 100 [55.0%] took pain medication within the last week and 44.0% (44/100) took pain medications every day (Table 3). Very few patients (5 of 99 [5.1%]) used home remedies or herbs for pain control. Among patients who took pain medications in the last week, most took nonopiate medications, including acetaminophen (22 of 55 [40.0%]) and diclofenac (24 of 55 [43.6%]), and only 12 took opiate medications, including morphine (7 of 55 [12.7%]) and tramadol (5 of 55 [9.1%]). Almost all participants (53 of 55 [96.4%]) understood how to take their pain medications correctly. However, only 5 of 54 participants (9.3%) reported complete improvement in their pain (Figure 2). Most participants (30 of 54 [55.6%]) felt that they needed a stronger pain medication, and 16 of 54 (29.6%) felt they needed to take more of the pain medication than what was prescribed.

**Table 3:** Use of pain medications

Pain medication variable	n (%)
Use of pain medication in the last week (n=100)	
No	45 (45.0)
Yes	55 (55.0)
Type of pain medication used (n=55)	
Paracetamol or Tylenol	22 (40.0)
Diclofenac	24 (43.6)
Morphine	7 (12.7)
Tramadol	5 (9.3)
How pain medications are taken (n=55)	
Scheduled	24 (43.6)
As needed	31 (56.4)
Understanding of how to take prescribed pain medications (n=55)	
No	2 (3.6)
Yes	53 (96.4)
Side effects experienced from pain medication (n=55)	
No	45 (81.8)
Yes	10 (18.2)
Use of pain medication every day in the last week (n=100)	
No	56 (56.0)
Yes	44 (44.0)
Use of home remedies or herbs for pain control (n=99)	
No	94 (95.0)
Yes	5 (5.1)

Bell. Barriers to cervical cancer pain control in urban Ghana. Am J Obstet Gynecol Glob Rep 2022.

**Figure 3:**
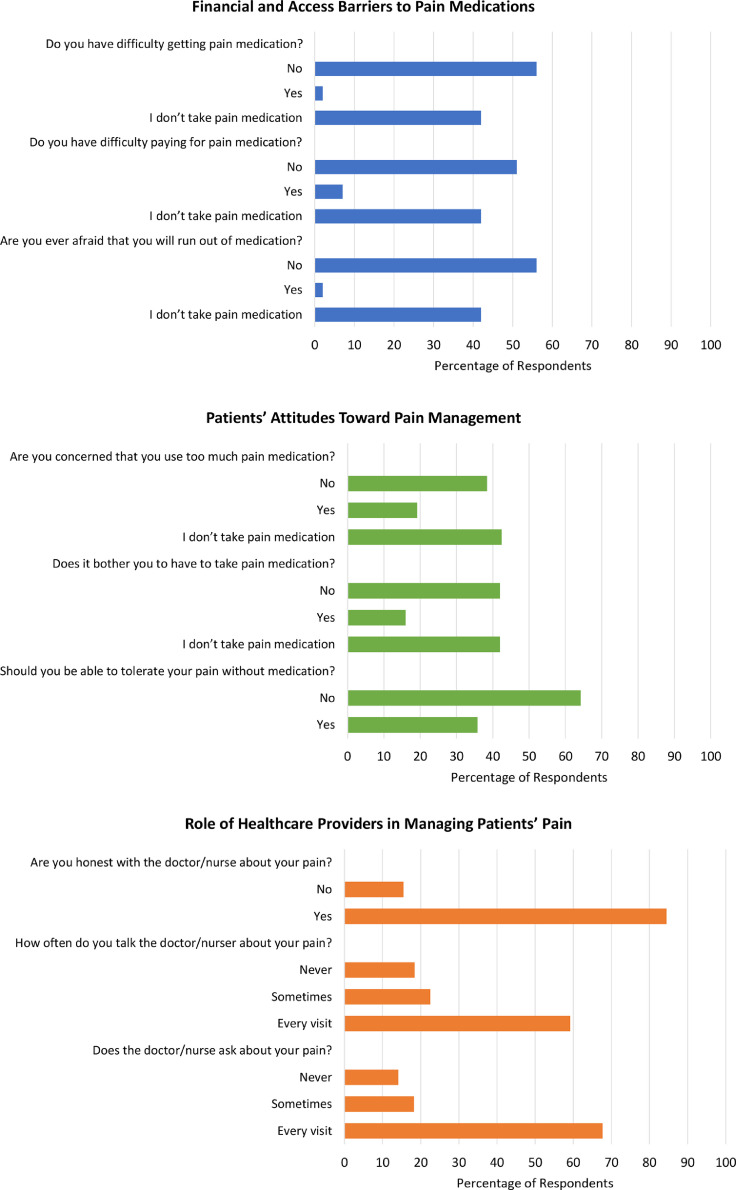
Factors contributing to unmet need for pain control

Figure 3 examines the factors contributing to patients’ unmet need for pain control. Most participants reported that their healthcare providers asked about their pain at every clinic visit (67 of 99 [67.7%]), discussed their pain at every clinic visit (58 of 98 [58.2%]), and were honest about their level of pain (82 of 97 [84.5%]). However, nearly one-seventh of participants (14 of 99 [14.1%]) reported that their healthcare providers never asked about their pain, they never discussed their pain with their provider (18 of 98 [18.4%]), and they did not honestly share their level of pain (15 of 97 [15.5%]). When asked about attitudes toward pain management, 34 of 95 participants (35.8%) thought that they should be able to tolerate their cervical cancer pain without medication. Among the 58 participants who reported ever taking pain medication for their cervical cancer pain, 16 (27.6%) were bothered that they took pain medication, and 19 (32.8%) were concerned that they used too much. Regarding financial and access barriers, most participants who reported ever taking a pain medication were able to afford the recommended medication (51 of 58 [87.9%]) and access (56 of 58 [96.6%]) the medication. In addition, 56 of 58 participants (97%) did not worry their supply would run out.

Bell. Barriers to cervical cancer pain control in urban Ghana. Am J Obstet Gynecol Glob Rep 2022.

A subanalysis of the 22 women who had pain but were not taking pain medications demonstrated that, for many, their pain was significant enough to negatively affect their daily activities (12 of 22 [54.5%]) and sleep (14 of 22 [63.6%]). However, 10 of 22 women (45.5%) did not talk about their pain at every clinic visit, and 11 of 22 women (50.0%) reported that their provider did not ask about pain at every clinic visit. Only 5 of 22 (22.7%) believed that women with cervical cancer should not take pain medications.

## Comment

### Principal findings

Among women with cervical cancer in Ghana, we demonstrated that most women had pain. Most ranked their pain as ≥3 on a 5-point pain scale and had pain significant enough to impact their sleep and ability to carry out daily activities. Barriers to adequate pain control included the healthcare provider's focus on pain, with nearly one-seventh of women reporting that their providers never asked about their pain and that they never discussed their pain with their provider. In addition, women's attitudes toward pain control played a role, with one-third of patients believing they should be able to tolerate their cervical cancer pain without medication and one-fourth being bothered that they take pain medication. Most participants were able to afford and access pain medications and did not worry their supply of medications would run out.

### Results

Consistent with the existing literature, we demonstrated an unmet need for pain control among women with cervical cancer in Ghana.^4^ Among our participants, 80% had pain from their cervical cancer, and more than half endorsed pain that was severe enough to limit their ability to complete activities of daily living. In 2020, the World Health Organization (WHO) identified cancer as the disease generating the most serious suffering and need for palliative care in LMICs, with the greatest worldwide need in Africa.^13^ Specific WHO guidelines on cervical cancer note that most women with cervical cancer will experience moderate to severe pain^13^ and that pain often persists after treatment is completed.^5^ Despite these studies indicating the importance of pain, a 2016 systematic review of literature on cervical cancer in Africa reported that quality of life among women with cervical cancer was only addressed in 4.1% of studies.^14^

The WHO identified barriers to effective pain relief in resource-limited areas, which include legislative and policy barriers, out-of-date national guidelines, inadequately trained healthcare providers, negative healthcare worker beliefs that are not evidenced based, and economic limitations.^1^ Patients in LMICs rarely have access to palliative care services for pain and symptom management because of the lack of providers and associated costs.^15^ Globally, the opioid epidemic has highlighted the potential dangers of opioid use and overuse and has altered current prescribing patterns. Consequences include both an increased focus on nonnarcotic pain regimens and provider hesitancy in prescribing opioids in high-income countries (HICs).^16^ Despite the current opioid epidemic in HICs,^16^ only 15% of low-income countries worldwide have adequate access to oral morphine analgesia compared with 77% of HICs,^17^ with particularly limited availability and accessibility in Africa.^18^ Unlike other studies done in LMICs,^7^ in our study, affordability and accessibility of pain medications were not significant barriers to women accessing pain medications. This difference may be partial because of our study site's urban location with the presence of multiple pharmacies. In addition, most patients in our study were taking nonopioid medications with generic options that are more available and affordable. If more patients in our study had been prescribed opioids, we anticipate that cost and accessibility barriers would have been higher. Despite the widespread use of traditional medicines in many parts of Africa,^19^ very few patients in our study reported using home remedies or herbs for pain control, possibly because of the urban study site or discomfort disclosing traditional medicine use because of the associated stigma.

Here, we found that the limited focus on pain control in patient-provider visits and patients’ attitudes toward pain management were barriers to adequate pain control. Regarding the role of healthcare providers in pain control, limited previous research has been done in LMICs, including sub-Saharan Africa. This may be because of African populations traditionally receiving palliative care from outside the formal healthcare system by family, local nonprofits, or community members.^18^^,^^20^ Studies done in HICs have demonstrated that providers often underestimate patients’ pain^21^—particularly that of female patients^22^—and that patient-provider communication about pain is essential.^23^ The existing literature is particularly sparse regarding patient perception of the use of medications to treat cervical cancer pain. Attitudes toward cervical cancer pain control may be influenced by both stigma surrounding cervical cancer and negative public perceptions about pain medication use.^24^ Many women in LMICs experience childbirth without analgesia, and complex cultural beliefs surrounding perceptions of pain, coping techniques, and attitudes toward pain control exist.^25^ These perceptions likely extend beyond childbirth and impact attitudes about cervical cancer pain.^26^

### Clinical implications

Our findings demonstrated key actionable gaps in healthcare delivery for women with cervical cancer in low-resource settings, such as Ghana, to address unmet needs for pain control. Our study highlighted the disconnect between clinical recommendations regarding frequent discussion of pain and actual practice of pain management, particularly in low-resource settings. Although most patients reported being honest with their provider regarding their pain, pain was not routinely discussed at every visit. Facilities that manage women with cervical cancer should integrate routine assessment of pain into every clinical encounter. This is especially important in LMIC settings where women are not traditionally involved in their healthcare and healthcare-related decisions and may not feel empowered to bring up topics that were not directly queried by their healthcare provider.^27^ In addition to asking about the presence and severity of pain, our findings demonstrated that clinical assessments should also address the impact pain has on sleep and daily activities. The assessment of pain severity is subjective,^28^ and the application of pain scales to LMIC settings may be challenged by limited patient numeracy. Incorporating assessments of how pain impacts concrete aspects of daily life may help women and their providers better identify unmet needs for pain control. Furthermore, consistent with the literature, women in our study had a high parity, and most women lived in poverty.^29^ As these women are typically the primary caretakers for children and grandchildren and perform most household tasks, cervical cancer pain can have large, unassessed consequences for the well-being of entire families.

Second, our findings demonstrated the importance and heterogeneity of patients’ perspectives on pain control and the use of pain medications. Despite a significant portion of our study population reporting uncontrolled pain because of their cancer, more than one-third of patients felt that they should be able to tolerate cervical cancer pain without medication, and one-fourth of patients were bothered that they took medication. Additional research is needed to further explore the complex relationships between attitudes toward pain and unmet needs for pain control. Furthermore, it is important that cultural beliefs are respected and women are not pressured to use unwanted medications—especially in the context of the opioid epidemic in HICs.^16^ To begin, measures should be taken to educate women with cervical cancer about the typical nature of pain associated with their cancer, locally available pain control options, and potential risks and benefits. Moreover, women should be educated on the changing role of pain as the cancer evolves and the potential impact of pain and other symptoms in tolerating recommended treatments. In facilities with a high volume of patients with cervical cancer, such as our study site, peer support groups could be used to share experiences with pain and pain control.

### Research implications

Our study identified a key subgroup of women who had pain from their cervical cancer yet were not taking pain medications. This paves the way for further studies, potentially qualitative, to further understand the complex personal and systems factors that create this gap in adequate pain control and to evaluate effective strategies to address it. In particular, further research is needed to understand the nuanced role of cost and whether nonusers of pain medication knew the costs of pain medications and whether that perceived cost was a driver of nonuse. Additional research is also needed to develop and validate a tool to define and assess unmet pain control needs in an LMIC setting, where lower numeracy and health literacy may limit the use of traditional scales. Recognizing the importance of locally specific factors, such as culture, availability of gynecology oncology prescribers, and availability of certain pain medications, multicountry studies could be conducted to understand the relative burdens of unmet pain needs.

### Strengths and limitations

The strengths of this study included the focus on pain and pain control among an understudied but important population: women with cervical cancer in an LMIC setting. However, this study has several limitations. First, it was conducted in a single country at a single, urban, tertiary care site, which may limit generalizability to other settings and other LMICs. This study site was selected because of the large volume of patients with cervical cancer and the presence of a focused gynecologic oncology division, which is rare in sub-Saharan Africa. Second, participants may have been hesitant to provide honest responses because they were actively receiving care for their cervical cancer at the same location where recruitment and data collection occurred. This is a particular concern for questions asking patients whether they honestly reported their pain to their providers. However, this issue was limited by ensuring an informed consent process, performing all research processes by a research assistant not involved in the patients’ clinical care, and not collecting any identifying information. Although many of the questions in our study were selected from a validated scale, some questions were specifically developed for this study with the expertise of local gynecologic oncologists. Finally, this study was limited by its sample size of 100 women. This was a considerable sample size given our focus on an intentionally narrow population of women with cervical cancer; however, it limited our ability to perform inferential statistics and meaningfully evaluate factors statistically associated with unmet needs for pain control.

### Conclusions

Pain because of cervical cancer continues to be a significant health issue globally, especially in low-resource settings, such as Ghana. Our study has offered a unique perspective from 100 patients undergoing treatment for cervical cancer. We found that most patients had pain because of their cervical cancer and that several patients were limited in their day-to-day activities because of pain. Our findings have challenged the assumption that financial and geographic barriers are the primary drivers of the unmet need for pain control and emphasize the importance of women's attitudes toward pain control and the role of healthcare providers in prioritizing pain management.
